# Endogenous Endophthalmitis Caused by *Prototheca* Microalga in Birman Cat, Spain

**DOI:** 10.3201/eid3101.241198

**Published:** 2025-01

**Authors:** Laura Jimenez-Ramos, Ana Ripolles-Garcia, Gianvito Lanave, Francesco Pellegrini, Miriam Caro-Suarez, Almudena Latre-Moreno, Marta Ferruz-Fernandez, Maria Luisa Palmero-Colado, Vanessa Carballes-Perez, Antonio Melendez-Lazo, Carolina Naranjo, Fernando Laguna, Vito Martella, Manuel Villagrasa

**Affiliations:** Puchol Veterinary Hospital, Madrid, Spain (L. Jimenez-Ramos, A. Ripolles-Garcia, M. Caro-Suarez, A. Latre-Moreno, M. Ferruz-Fernandez, F. Laguna, M. Villagrasa); Centro Oftalmológico Veterinario Goya, Madrid (L. Jimenez-Ramos, A. Ripolles-Garcia, M. Caro-Suarez, A. Latre-Moreno, M. Ferruz-Fernandez, F. Laguna, M. Villagrasa); University of Bari Aldo Moro, Bari, Italy (G. Lanave, F. Pellegrini, V. Martella); Gattos Veterinary Hospital, Madrid (M.L. Luisa Palmero-Colado, V. Carballes-Perez); T-Cito Laboratories, Barcelona, Spain (A. Melendez-Lazo); IDEXX Laboratories, Barcelona (C. Naranjo); University of Veterinary Medicine, Budapest, Hungary (V. Martella)

**Keywords:** endophthalmitis, feline, protothecosis, zoonoses, *Prototheca*, microalga, alga, algae, Spain, Birman

## Abstract

We identified *Prototheca* spp. microalga in ocular samples of a cat in Spain with nontreatable endogenous endophthalmitis. Within 2 years, the eye lesions progressively worsened and neurologic signs appeared, suggesting systemic spread of the infection. On multitarget sequence analysis, the feline pathogen could not be assigned to any known *Prototheca* species.

Protothecosis is an uncommon disease caused by the unicellular microalga *Prototheca* spp., described in humans and animals and associated with systemic disease, cutaneous lesions, or both (*1*,*2*). *Prototheca* spp. has been identified as the cause of cutaneous lesions and in 1 case of disseminated neurologic disease in cats (*2*–*4*). Diagnosis of protothecosis can be challenging and usually is based on observation of the organism in tissues and body fluids (*5*). Culturing or PCR is required for a definitive diagnosis and species identification (*2*,*4*).

A 5-year-old female Birman cat, spayed and maintained indoors, was referred to our veterinary hospital for a 1.5-month history of uveitis in the right eye. Neuro-ophthalmic evaluation revealed that the right eye was blind and had severe signs of uveitis, whereas the left eye was unaffected. Ultrasound examination showed exudative retinal detachment in the right eye, confirming irreversible blindness. At 5.5 months, clinical signs of uveitis appeared also in the other eye (Figure, panel A). At the 6.5-month follow-up, the aqueous flare in the left eye worsened (Figure, panel B). We obtained an aqueous humor sample and a vitreous sample for cytologic examination, which revealed a mixed inflammatory process and the presence in the vitreous of structures morphologically compatible with algae of the genus *Prototheca* spp. (Appendix Figure 1, panel A). Antifungal therapy with itraconazole (5 mg/kg 2×/d) was initiated at 10.5 months and stopped voluntarily by the owner at 14.5 months. At the 16.5-month follow-up, the cat was completely blind, and the clinical signs had worsened with the development of corneal macro-deposits (Figure, panels C, D). At 17.5 months, we observed 2 episodes of neurologic clinical signs, including vestibular signs, ataxia, and disorientation. At 19.5 months, the owner reported that the cat had lost her hearing. At 21.5 months, the cat’s neurologic status further deteriorated, with the onset of seizures and prolonged anorexia. At this point, the owner opted for the humane euthanasia of the cat but did not give consent for a full-body necropsy.

**Figure:**
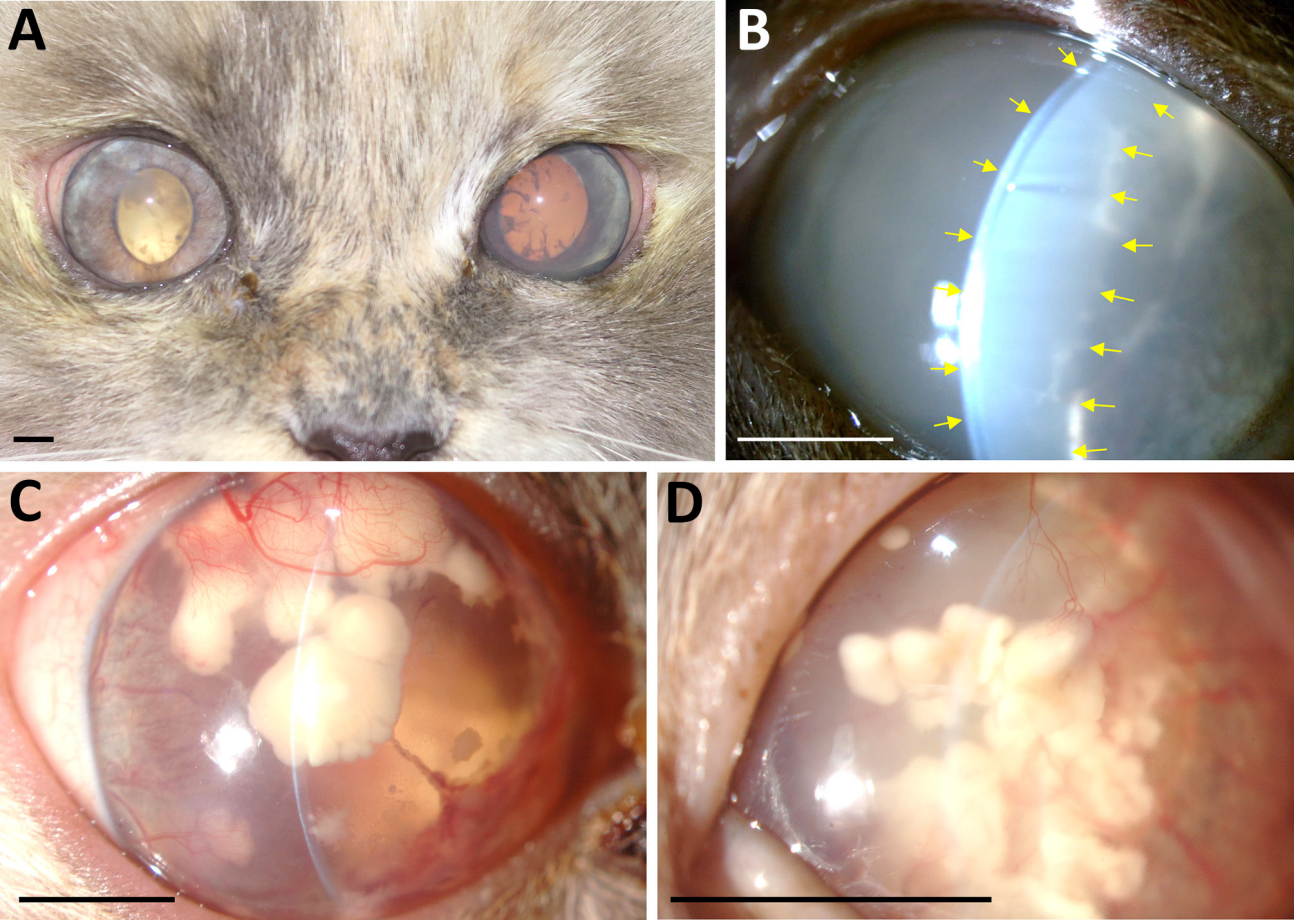
Clinical course of bilateral endogenous endophthalmitis in 5-year-old female Birman cat evaluated by slit-lamp biomicroscopic examination, Madrid, Spain. A) Digital photograph of both eyes demonstrating bilateral uveitis at 5.5 months after initial visit to clinic. B) Slit-lamp biomicroscopic image (original magnification ×10) of the left eye, demonstrating a marked flare (yellow arrows) at 6.5 months after initial clinical signs. C, D) At 16.5 months, the right eye (C) (original magnification ×10) and left eye (D) (original magnification ×16) were imaged by slit-lamp biomicroscopic examination, revealing corneal endothelial macrodeposits of undefined origin, presumed to be the result of *Prototheca* spp. invasion. Scale bars indicate 5 mm.

The eyes of the cat were submitted for biopsy. The samples had been frozen before fixation and autolysis had occurred, so histopathologic investigations were challenging because of artifacts. Nevertheless, diffuse exudate was visible throughout all the ocular structures (Appendix Figure 1, panel B). We observed karyorrhectic remnants and microorganisms within the axial cornea (Appendix Figure 1, panel C). In addition, the lens capsule was ruptured, and we noted the presence of intra-lenticular microorganisms and hypermature cataract formation (Appendix Figure 1, panel D). The microorganisms exhibited a markedly periodic acid-Schiff–positive and Alcian blue–negative membrane. Results of PCR analysis for *Cryptococcus* spp. were negative and, on the basis of the morphology and staining characteristics, we suspected a diagnosis of *Prototheca* spp. infection.

The ocular samples tested positive for *Prototheca* spp. in PCR tests that used 3 primer sets (Appendix Table 1) and amplified a 1,800-bp sequence of the 18S rDNA, a 630-bp sequence of 28S rDNA, and a 650-bp sequence of the cytochrome B gene. We deposited the nucleotide sequences in GenBank (accession nos. PQ111814 [18S rDNA sequence], PQ122806 [28S rDNA sequence], and PQ115153 [cytochrome B gene sequence]). We conducted multitarget sequence and phylogenetic analysis by using the sequences generated in this study and cognate sequences retrieved from the National Center for Biotechnology Information database (Appendix Figure 2). The 18S rDNA, the 28S rDNA, and the cytochrome B gene sequences shared the highest nucleotide identity with *Prototheca lentecrescens* PK1 (GenBank accession nos. MZ198751 [86.0%], OK236514 [84.8%], and MW701399 [83.5%]) (Appendix Table 2). The feline *Prototheca* strain was segregated in a separate branch within the maximum-likelihood phylogenetic tree, diverging from other *Prototheca* species, thereby supporting the hypothesis of a distinct phylotaxonomic status for the strain SPA/2024/cat/259 (Appendix Figure 2).

Disseminated *Prototheca* infection already has been reported in a cat with central nervous system signs and a suspected diagnosis of multifocal lymphoma; however, in other reports, feline protothecosis has been associated with cutaneous or subcutaneous lesions (*2*–*4*). In our case, the cat had a history of chronic glucocorticoid administration for intestinal disease, which probably triggered immune suppression in the animal. In addition, the cat had received 2 fecal transplants, which might be a potential source of infection. Also, previous studies have indicated that the Birman breed is highly susceptible to certain infectious diseases, including chlamydophilosis, cryptococcosis, feline infectious peritonitis, and *Tritrichomonas fetus* infection (*6*–*9*). However, we could not identify the primary source of the infection because this microalga can be found in multiple environmental sources. Another limitation of our study was that we could not isolate the *Prototheca* strain in vitro to assess its cultural properties. 

In conclusion, we describe a novel candidate *Prototheca* species invading the ocular tissues of a cat, a rare clinical manifestation in felids. Our findings also extend the knowledge of the genetic diversity of *Prototheca* spp. in animals, a piece of valuable information for pathogens with zoonotic potential.

Appendix Additional information about endogenous endophthalmitis caused by *Prototheca* microalga in cat, Spain.
