# Supplementary material for: Endogenous Endophthalmitis Caused by Prototheca Microalga in Birman Cat, Spain
Source: Emerg Infect Dis. 2025 Jan;31(1):189–92. doi: 10.3201/eid3101.241198 (PMC11682826; doi:10.3201/eid3101.241198)
Supplement: Appendix — Additional information about endogenous endophthalmitis caused by Prototheca microalga in cat, Spain. [file 24-1198-Techapp-s1.pdf]

Article DOI: https://doi.org/10.3201/eid3101.241198

EID cannot ensure accessibility for supplementary materials supplied by authors. Readers who have difficulty accessing supplementary content should contact the authors for assistance.

Endogenous Endophthalmitis Caused by a *Prototheca* Microalga in a Birman Cat, Spain

Appendix

Appendix Table 1. List of oligonucleotides used in this study

Target	Primer/Probes	Sequence 5′→3′	Reference
18S rDNA	SSUF1	AACCTGGTTGATCCTGCCAGTAGTC	(*1*)
	SSUR1	TGATCCTTCTGCAGGTTCACCTACG	
28S rDNA	28SF1	AAGCATATCAATAAGCGGAGG	(*2*)
	635	GGTCCGTGTTTCAAGACGG	
Cytochrome B	cytB_F1	GYGTWGAACAYATTATGAGAG	(*3*)
	cytB_R2	WACCCATAARAARTACCATTCWGG	

Appendix Table 2. Sequence analysis of the 18S rDNA ribosomal subunit, D1/D2 region of 28S rDNA ribosomal subunit and partial cytochrome B gene. Interrogation of NCBI databases (accessed July 2024) was carried out using the Basic Local Alignment Search Tool (BLAST)

	% nt identity by NCBI Blast					
	18S rDNA SSU		28S rDNA LSU		Cytochrome B	
Strain	% nt identity	Ref (PQ111814)	% nt identity	Ref (PQ122806)	% nt identity	Ref (PQ115153)
SPA/2024/cat/259	86.0	*P. lentecrescens* PK1 (MZ198751)	84.8	*P. lentecrescens* PK1 (OK236514)	83.5	*P. lentecrescens* PK1 (MW701399)

References

1. Xiao L, Morgan UM, Limor J, Escalante A, Arrowood M, Shulaw W, et al. Genetic diversity within *Cryptosporidium parvum* and related *Cryptosporidium* species. Appl Environ Microbiol. 1999;65:3386–91. PubMed https://doi.org/10.1128/AEM.65.8.3386-3391.1999

2. Ebihara M, Makimura K, Sato K, Abe S, Tsuboi R. Molecular detection of dermatophytes and nondermatophytes in onychomycosis by nested polymerase chain reaction based on 28S ribosomal RNA gene sequences. Br J Dermatol. 2009;161:1038–44. PubMed https://doi.org/10.1111/j.1365-2133.2009.09249.x

3. Jagielski T, Gawor J, Bakuła Z, Decewicz P, Maciszewski K, Karnkowska A. *cytb* as a new genetic marker for differentiation of *Prototheca* species. J Clin Microbiol. 2018;56:e00584–18. PubMed https://doi.org/10.1128/JCM.00584-18

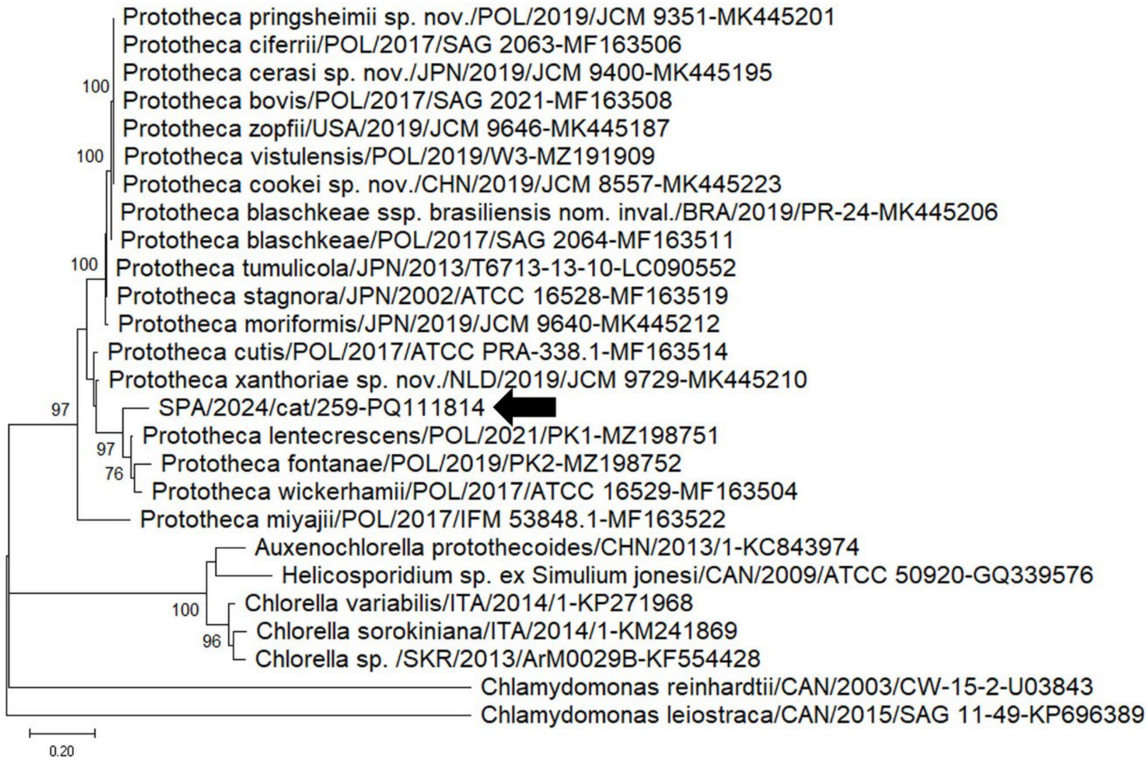

Appendix Figure 1. Unrooted phylogenetic tree. The sequences were derived from the complete 18S rDNA (1800 nt) from the species of the candidate *Prototheca* described in this study and related reference strains retrieved from the GenBank database. The phylogeny was constructed using the maximum-likelihood (ML) method, the general time-reversible (GTR) model with a gamma distribution and invariable sites to model evolutionary rate differences among sites (6 categories). The robustness of the nodes on the phylogenetic tree was evaluated through the application of 1,000 bootstrap replicates. Bootstrap values exceeding 75% are indicated. The black arrows indicate the lineage identified in the present study. The scale bars indicate the estimated number of nucleotide substitutions.

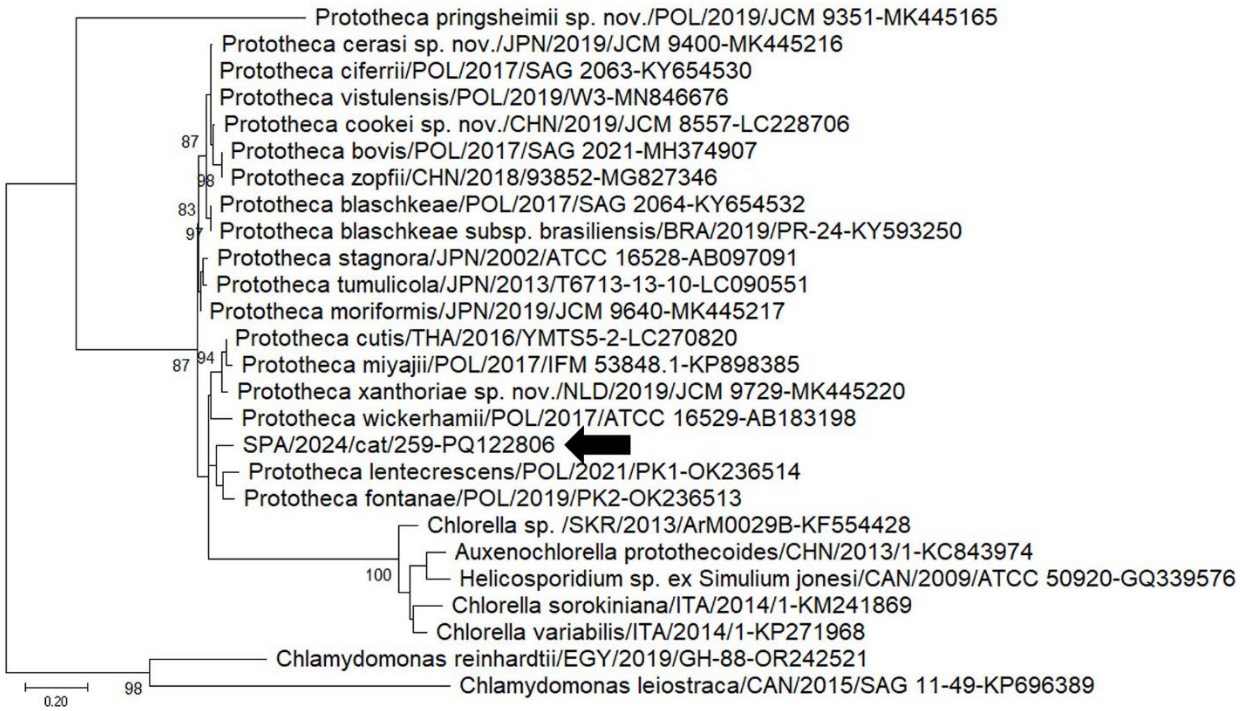

Appendix Figure 2. Unrooted phylogenetic tree. The sequences were derived from the partial 28S rDNA (630 nt) from the species of the candidate *Prototheca* described in this study and related reference strains retrieved from the GenBank database. The phylogeny was constructed using the maximum-likelihood (ML) method, the general time-reversible (GTR) model with a gamma distribution and invariable sites to model evolutionary rate differences among sites (6 categories). The robustness of the nodes on the phylogenetic tree was evaluated through the application of 1,000 bootstrap replicates. Bootstrap values exceeding 75% are indicated. The black arrows indicate the lineage identified in the present study. The scale bars indicate the estimated number of nucleotide substitutions.

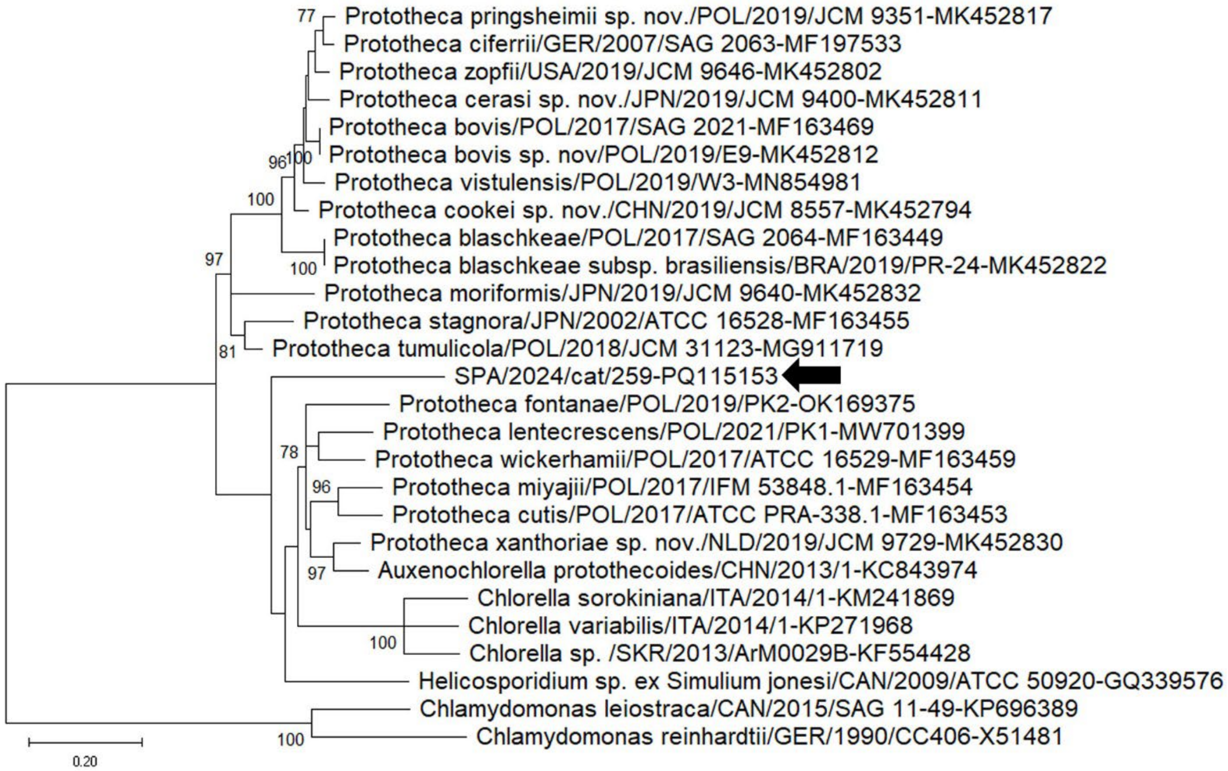

Appendix Figure 3. Unrooted phylogenetic tree. The sequences were derived from the partial cytochrome B gene (650 nt) from the species of the candidate *Prototheca* described in this study and related reference strains retrieved from the GenBank database. The phylogeny was constructed using the maximum-likelihood (ML) method, the general time-reversible (GTR) model with a gamma distribution and invariable sites to model evolutionary rate differences among sites (6 categories). The robustness of the nodes on the phylogenetic tree was evaluated through the application of 1,000 bootstrap replicates. Bootstrap values exceeding 75% are indicated. The black arrows indicate the lineage identified in the present study. The scale bars indicate the estimated number of nucleotide substitutions.

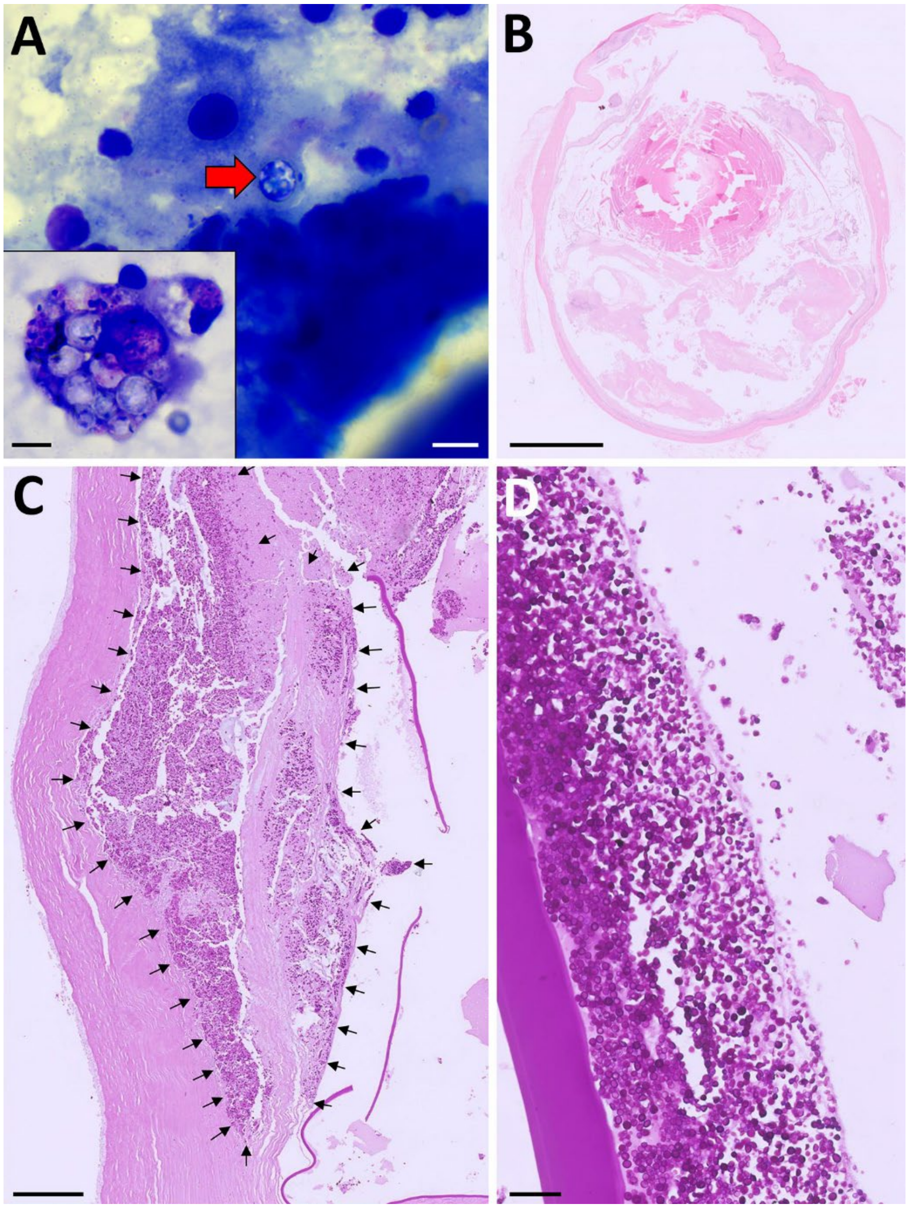

Appendix Figure 4. Cytologic and histologic evaluation of vitreal samples and ocular tissues. A. Modified Romanowsky staining of vitreal centesis sample reveals the presence of numerous, rounded microorganisms measuring 5–6 μm in diameter with a thin capsule, compatible with algae of the genus *Prototheca* spp. (red arrow). The inset illustrates the presence of approximately ten microorganisms phagocytosed by a macrophage. Scale bars: 20μm. B. Whole eye section stained with hematoxylin and eosin demonstrates severe artifacts resulting from autolysis, as well as the presence of eosinophilic exudates filling the anterior and posterior chambers, and the posterior segment. Scale bar: 5mm. C. Use of periodic acid-Schiff (PAS) staining to reveal the presence of multiple microorganisms invading the deep corneal stroma corresponding to the previously detected macrodeposits (black arrows). Scale bar: 200μm. D. Magnification section of the PAS-stained lens demonstrating the presence of multiple intralenticular microorganisms. Scale bar: 200μm.
